# Thrombolysis in Cerebral Infarction (TICI) 2b Versus TICI 3 Reperfusion in Stroke Patients with Mild Symptoms

**DOI:** 10.1007/s00062-026-01631-x

**Published:** 2026-03-13

**Authors:** Christian Heitkamp, Uta Hanning, Alexander Heitkamp, Luca Meucci, Susan Klapproth, Felix Schlicht, Gabriel Broocks, Tobias D Faizy, Helge C. Kniep, Fabian Flottmann, Maxim Bester, Maximilian Schell, Christian Thaler, Götz Thomalla, Jens Fiehler, Laurens Winkelmeier, German Stroke Registry – Endovascular Treatment (GSR-ET)

**Affiliations:** 1 Department of Neuroradiology, University Medical Center Hamburg-Eppendorf, Hamburg, Germany; 2 Department of Neuroradiology, University Medical Centre Mannheim, Mannheim, Germany; 3 Department of Neuroradiology, HELIOS Medical Center, Schwerin, Germany; 4 Department of Neuroradiology, University Hospital Münster, Münster, Germany; 5 Department of Neurology, University Medical Center Hamburg-Eppendorf, Hamburg, Germany

**Keywords:** Stroke, Angiography, Mechanical thrombectomy, Minor stroke, Reperfusion status

## Abstract

**Purpose:**

The treatment effect of mechanical thrombectomy in patients with mild stroke symptoms and large vessel occlusion remains unclear. Furthermore, it is uncertain whether achieving complete reperfusion (modified Thrombolysis in Cerebral Infarction [mTICI] 3) offers additional clinical benefit compared to incomplete reperfusion (mTICI 2b) in patients with mild stroke.

**Methods:**

In this retrospective, multicenter analysis, all patients enrolled in the German Stroke Registry—Endovascular Treatment between 2015 and 2023 (*n* = 18,069) were evaluated. The study included patients with a National Institutes of Health Stroke Scale (NIHSS) score of < 6 and anterior circulation large vessel occlusion. Clinical outcomes were compared between mTICI 2b and mTICI 3. The primary endpoint was excellent functional outcome at 90 days, defined as a modified Rankin Scale (mRS) score of 0–1.

**Results:**

Of the 747 included patients, 37% achieved mTICI 2b and 63% mTICI 3. An excellent functional outcome was observed at similar rates in both groups (mTICI 2b: 59% vs. mTICI 3: 60%; *P* = 0.83; adjusted OR: 0.96 [95% CI: 0.66–1.40]; *P* = 0.84). The rates of symptomatic intracranial hemorrhage (4% vs. 3%; *P* = 0.36) and 90-day mortality (10% vs. 7%; *P* = 0.26; adjusted OR: 0.80 [95% CI: 0.42–1.50]; *P* = 0.49) were also comparable between the two groups.

**Conclusion:**

Among thrombectomy patients with mild stroke, complete reperfusion was not associated with better functional outcomes than incomplete reperfusion. These findings suggest that mTICI 2b may be an acceptable final angiographic result and raise questions about the risk-benefit ratio of additional retrieval maneuvers aimed at achieving mTICI 3.

**Supplementary Information:**

The online version of this article (https://doi.org/10.1007/s00062-026-01631-x) contains supplementary material, which is available to authorized users.

## Introduction

Despite lacking randomized controlled evidence, mechanical thrombectomy (MT) is frequently performed in acute ischemic stroke patients with large vessel occlusion who present with only mild stroke symptoms [1–4]. Pending the results from the ongoing randomized trials ENDOLOW [Endovascular Therapy for Low NIHSS Ischemic Strokes] [5] and MOSTE [Minor Stroke Therapy Evaluation] [6], current guidelines recommend an individualized approach to endovascular therapy in these patients [7, 8].

The modified Thrombolysis in Cerebral Infarction (mTICI) scale is widely accepted as the standard metric for assessing the technical success of MT. It quantifies the degree of tissue reperfusion based on the angiographic appearance of the initially occluded target artery territory following endovascular therapy [9]. The mTICI scale ranges from no reperfusion (mTICI 0) to complete reperfusion (mTICI 3) [9]. Previous studies on patients with more severe ischemic stroke symptoms have indicated that complete reperfusion (mTICI 3) is associated with more favorable functional outcomes than incomplete reperfusion (mTICI 2b), mainly owing to smaller infarct progression [10, 11]. In line with these findings, the European Stroke Organisation—European Society for Minimally Invasive Neurological Therapy (ESO–ESMINT) guidelines advocate achieving mTICI 3 reperfusion, if achievable with reasonable safety [7].

However, a key difference in patients with minor stroke lies in the presence of more favorable arterial collaterals and the overall smaller tissue-at-risk volumes [12, 13]. Given the uncertain risk–benefit ratio of MT in this patient population, we hypothesized that achieving mTICI 2b reperfusion may represent an acceptable outcome in patients with minor stroke.

## Materials and Methods

### Study Design and Participating Centers

This retrospective multicenter cohort study included patients prospectively enrolled in the German Stroke Registry–Endovascular Treatment (GSR-ET) between 2015 and 2023. The GSR-ET is an ongoing, open-label, multicenter registry comprising patients who underwent MT at 25 comprehensive stroke centers in Germany (ClinicalTrials.gov identifier: NCT03356392). Adults (≥ 18 years) with acute ischemic stroke due to large vessel occlusion who received MT are included by each participating site.

Ethical approval was obtained from the Ludwig Maximilian University, Munich, Germany (689-15), and from local ethics committees of all participating centers. Informed consent for this study was waived after review of the ethics committee of each participating center. The study followed the Strengthening the Reporting of Observational Studies in Epidemiology (STROBE) guidelines [14] and adhered to the principles of the Declaration of Helsinki and the Health Insurance Portability and Accountability Act (HIPAA).

### Study In- and Exclusion Criteria

The inclusion criteria for this study were: (1) patients with acute ischemic stroke due to an isolated occlusion of the intracranial internal carotid artery (ICA) or the M1 or M2 segment of the middle cerebral artery; (2) a baseline National Institutes of Health Stroke Scale (NIHSS) score of < 6; (3) successful reperfusion as defined by an mTICI score of 2b–3; and (4) complete data on baseline NIHSS, prestroke modified Rankin Scale (mRS), final mTICI grade, and mRS at 90 days. Patients with a prestroke mRS of ≥ 2 were excluded. A detailed illustration of the in- and exclusion criteria is shown in Supplementary Fig. 1.

### Clinical and Radiological Data Acquisition

Patient characteristics, radiological parameters, and functional outcomes were extracted from the GSR-ET. Baseline imaging, digital subtraction angiograms, and follow-up imaging were assessed by local investigators at each participating center. The reperfusion status was determined by the treating neurointerventionalist using the mTICI scale (without rating of mTICI 2c) [15]. Successful reperfusion was defined as a final mTICI score of 2b–3. Clinical status was evaluated at admission, 24 h, and 90 days after MT using the NIHSS and the mRS.

### Outcome Measures

The primary outcome was an excellent functional outcome, defined as an mRS score of 0–1 at 90 days after treatment [1, 16]. Secondary outcomes included functional independence (mRS 0–2) at 90 days, an ordinal shift across the mRS at 90 days and the NIHSS after 24 h. Safety outcomes comprised symptomatic intracranial hemorrhage (sICH), defined according to European Cooperative Acute Stroke Study II (ECASS II) criteria as any intracranial hemorrhage within 24 h associated with a ≥ 4-point increase on the NIHSS, [17] and all-cause mortality within 90 days after stroke.

### Statistical Analysis

Descriptive statistics were used to compare mTICI 2b and mTICI 3 groups. Continuous variables were analyzed using the Mann–Whitney U test and categorical variables with the χ^2^ test. Normality of data distribution was verified with the Shapiro–Wilk test. Continuous variables are presented as medians with interquartile ranges (IQR), and categorical variables as counts and percentages. Different regression models were employed depending on the scale of the outcome variables. Multivariable logistic regression was used for binary outcomes, including excellent functional outcome (mRS 0–1), functional independence (mRS 0–2), and mortality at 90 days, with results reported as adjusted odds ratios (aOR). For the 90-day mRS score as an ordinal outcome, a multivariable ordinal logistic regression was performed to estimate the adjusted common odds ratio; the proportional odds assumption was verified using the Brant test (*P* = 0.46). Finally, a multivariable linear regression was used to analyze NIHSS scores at 24 h, with results reported as adjusted beta coefficients. Logistic and linear regression models were adjusted for age, sex, baseline NIHSS, time from symptom onset/last seen well to admission, Alberta Stroke Program Early CT Score (ASPECTS), occlusion location (ICA/M1 vs. M2), administration of tissue plasminogen activator and anesthetic regimen. The variables were selected a priori based on their clinical relevance and evidence from previous studies. Multivariable analyses were conducted using complete case analysis. The full model of the multivariable logistic regression analysis for the primary outcome can be found in Supplementary Table 1. To adjust for procedural complexity, we conducted a sensitivity analysis using a multivariable model that included the number of passes as a dichotomized variable (1–2 vs. ≥ 3 attempts; Supplementary Table 2). Statistical significance was defined as *P* < 0.05. All analyses were performed using Stata (Stata/MP18, StataCorp, TX, USA).

## Results

### Baseline Characteristics

Following the inclusion criteria, a total of 747 patients with minor stroke symptoms who achieved successful reperfusion (mTICI 2b or 3) were included in the analysis. Among these, 37% achieved mTICI 2b and 63% mTICI 3 (Table 1). Across all patients, the median age was 70 (IQR, 61–80) and 48% were female. The median NIHSS score at admission was 4 (IQR, 2–5) and the median time from symptom onset/last seen well to admission was 179 min (IQR, 78–400). With regards to treatment characteristics, 44% of patients received intravenous tissue plasminogen activator (tPA) and the median time from admission to groin puncture was 82 min (IQR, 55–130).

**Table 1:** Patients’ baseline, procedural and outcome characteristics

	Total	mTICI 2b	mTICI 3	*P*
	*N* = 747	*N* = 276	*N* = 471	
*Baseline characteristics*				
Age	70 (61–80)	70 (60–80)	71 (61–79)	0.74
Male sex	388 (52%)	146 (53%)	242 (51%)	0.69
Prestroke mRS	0 (0–0)	0 (0–0)	0 (0–0)	0.76
NIHSS at admission	4 (2–5)	4 (2–5)	3 (2–5)	0.14
Hypertension	545 (73%)	192 (70%)	353 (75%)	0.11
Diabetes mellitus	143 (19%)	46 (17%)	97 (21%)	0.19
Dyslipidemia	326 (44%)	105 (38%)	221 (47%)	0.019
Atrial fibrillation	251 (34%)	86 (31%)	165 (35%)	0.28
Antithrombotic medication	203 (27%)	67 (25%)	136 (29%)	0.18
Anticoagulant medication	82 (11%)	22 (8%)	60 (13%)	0.045
Time from symptom onset/last seen well to admission (min)	179 (78–400)	180 (77–408)	177 (80–398)	0.83
*Imaging and occlusion site*				
ASPECTS	9 (8–10)	9 (8–10)	10 (8–10)	< 0.001
Intracranial ICA	107 (14%)	43 (16%)	64 (14%)	0.45
M1	337 (45%)	115 (42%)	222 (47%)	0.15
M2	303 (41%)	118 (43%)	185 (39%)	0.35
*Treatment characteristics*				
Administration of tPA	326 (44%)	129 (47%)	197 (42%)	0.18
General anesthesia	487 (67%)	159 (60%)	328 (71%)	0.004
Time from admission to groin puncture (min)	82 (55–130)	82 (57–135)	82 (53–127)	0.76
Time from groin puncture to flow restoration (min)	40 (25–64)	49 (30–70)	34 (24–59)	< 0.001
Time from admission to flow restoration (min)	133 (95–197)	144 (102–199)	128 (91–197)	0.053
No. of passes	1 (1–3)	2 (1–3)	1 (1–2)	< 0.001
Adverse event during treatment	131 (18%)	57 (21%)	74 (16%)	0.078
Vasospasm	39 (5%)	20 (7%)	19 (4%)	0.057
Clot migration and embolization	28 (4%)	15 (5%)	13 (3%)	0.063
Dissection or perforation	20 (3%)	10 (4%)	10 (2%)	0.22

Data are presented as median (IQR) for continuous measures, and *n* (%) for categorical measures. Characteristics were compared by using either Mann-Whitney U test for continuous variables or a chi-square test for categorical variables. Statistical significance: *p* < 0.05.

*mTICI* modified Thrombolysis in Cerebral Infarction; *NIHSS* National Institutes of Health Stroke Scale; *ICA* Internal Carotid Artery; *M1/M2* M1 or M2 segment of the middle cerebral artery; *ASPECTS* Alberta Stroke Program Early CT score; *tPA* tissue plasminogen activator; *mRS* modified Rankin Scale

Compared to patients who achieved mTICI 3 reperfusion, patients with mTICI 2b reperfusion had lower ASPECTS at admission (median 9 vs. 10; *P* < 0.001) and underwent less often general anesthesia (60% vs. 71%; *P* = 0.004). Time interval from groin puncture to flow restoration was longer (median 49 vs. 34 min; *P* < 0.001), and a higher number of passes was conducted (median 2 vs. 1; *P* < 0.001) to achieve successful reperfusion. There were no significant differences in adverse events during treatment between an mTICI 2b and mTICI 3 reperfusion.

### Functional and Safety Outcomes

**Fig. 1:**
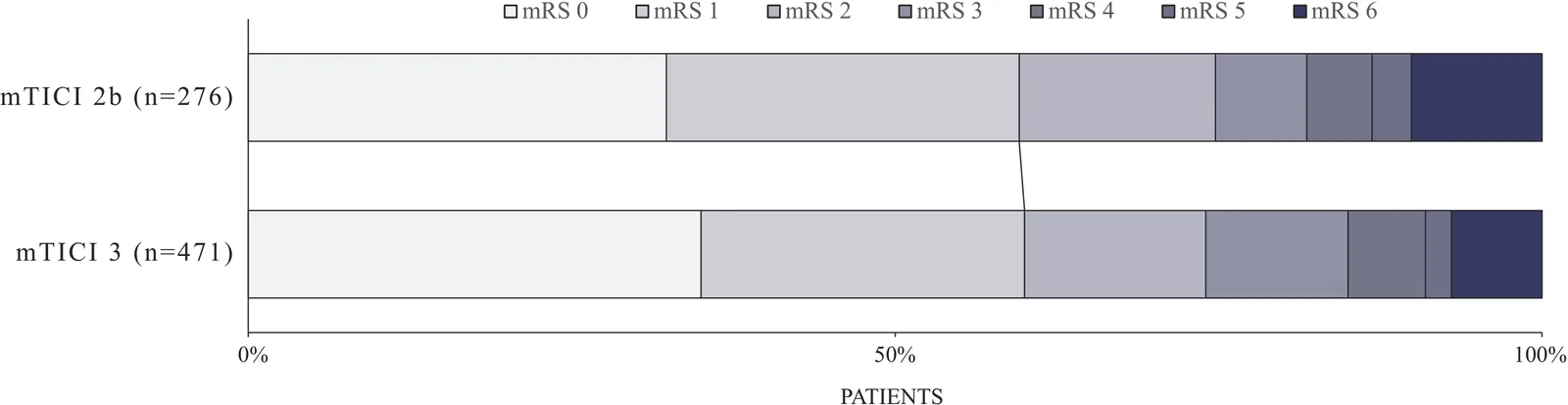
Distribution of the modified Rankin Scale (mRS) score stratified by the final angiographic result. The mRS ranges from 0 to 6 and assesses functional disability after stroke, with 0 indicating no symptoms and 6 indicating death. The modified Thrombolysis in Cerebral Infarction (mTICI) score measures reperfusion after mechanical thrombectomy; mTICI 2b indicates incomplete reperfusion, while mTICI 3 reflects complete reperfusion. An excellent functional outcome (mRS 0–1; straight line) was achieved at similar rates in patients with mTICI 2b and mTICI 3 reperfusion (59% vs. 60%; *P* = 0.83; adjusted OR: 0.96 [95% CI: 0.66–1.40], *P* = 0.84)

An excellent functional outcome was observed at similar rates in both reperfusion groups (Fig. 1, mTICI 2b: 59% vs. mTICI 3: 60%; *P* = 0.83; adjusted OR: 0.96 [95% CI: 0.66–1.40], *P* = 0.84). Likewise, there was no significant difference between an mTICI 2b and mTICI 3 reperfusion with regards to functional independence (74% vs. 74%; *P* = 0.91; adjusted OR: 0.98 [95% CI: 0.64–1.50], *P* = 0.93; Table 2) as well as 90-day mRS scores (median 1 vs. 1; *P* = 0.52; adjusted common OR: 0.90 [95% CI: 0.66–1.24], *P* = 0.53). The NIHSS score after 24 h was higher in patients with mTICI 2b reperfusion (median 3 vs. 2; *P* = 0.049); however, there was no significant association after adjustment in regression analysis (adjusted beta coefficient: −0.64 [95% CI: −1.80–0.52], *P* = 0.28).

**Table 2:** Functional and safety outcomes stratified by final reperfusion result (mTICI 2b vs. mTICI 3) in patients with minor stroke.

	Patients, No.(%)				Odds Ratio (95% Confidence interval)			
	Total (*n* = 747)	mTICI 2b (*n* = 276)	mTICI 3 (*n* = 471)	*P* ^a^	Unadjusted ^b^	*P*	Adjusted ^c^	*P*
*Primary Outcome*								
Excellent functional outcome [90-day mRS score 0–1]	445 (60%)	163 (59%)	282 (60%)	0.83	1.03 (0.76–1.40)	0.827	0.96 (0.66–1.40)	0.844
*Secondary Outcomes*								
Functional independence [90-day mRS score 0–2]	553 (74%)	205 (74%)	348 (74%)	0.91	0.98 (0.70–1.38)	0.907	0.98 (0.64–1.50)	0.930
90-day mRS score, median (IQR)	1 (0–3)	1 (0–3)	1 (0–3)	0.52	0.92 (0.70–1.20) ^d^	0.524	0.90 (0.66–1.24) ^d^	0.528
NIHSS after 24 h, median (IQR)	2 (1–5)	3 (1–6)	2 (1–5)	0.049	−0.41 (−1.52–0.69) ^e^	0.463	−0.64 (−1.80–0.52) ^e^	0.280
*Safety Outcomes*								
sICH within 24 h	24 (3%)	11 (4%)	13 (3%)	0.36	0.68 (0.30–1.55)	0.362	−^f^	
Mortality within 90 days	62 (8%)	27 (10%)	35 (7%)	0.26	0.74 (0.44–1.25)	0.262	0.80 (0.42–1.50)	0.485

^a^ Data are presented as *n* (%) for all categorical measures. Characteristics were compared by using a chi-square test for categorical variables and Mann-Whitney U test for continuous variables. Statistical significance: *p* < 0.05.

^b^ Univariable regression analysis with final reperfusion result (mTICI 3 vs. mTICI 2b) as independent variable. Reference level: mTICI 2b.

^c^ Multivariable regression analysis. Reference level: mTICI 2b. Results were adjusted for age, sex, baseline NIHSS, time from symptom onset/last seen well to admission, ASPECTS, occlusion location (ICA/M1 vs. M2), administration of tissue plasminogen activator and anesthetic regimen.

^d^ Adjusted common OR were calculated from ordinal logistic regression analysis. Values lower than 1 indicate lower odds of a shift in the distribution of 90-day mRS scores towards higher values (worse functional outcomes) favoring mTICI 3 compared to mTICI 2b.

^e^ Multivariable linear regression. Reference level: mTICI 2b. Results were adjusted for age, sex, baseline NIHSS, time from symptom onset/last seen well to admission, ASPECTS, occlusion location (ICA/M1 vs. M2), administration of tissue plasminogen activator and anesthetic regimen.

^f^ Due to the limited number of cases with symptomatic intracranial hemorrhage (*n* = 24), we did not perform a multivariable logistic regression.

*mTICI* modified Thrombolysis in Cerebral Infarction; *mRS* modified Rankin Scale; *NIHSS* National Institutes of Health Stroke Scale; *sICH* symptomatic Intracranial Hemorrhage; *ASPECTS* Alberta Stroke Program Early CT Score; *ICA* Internal Carotid Artery; *M1/M2* M1 or M2 segment of the middle cerebral artery

There were no significant differences between mTICI 2b and mTICI 3 reperfusion regarding the rates of sICH within 24 h (4% vs. 3%; *P* = 0.36) and mortality within 90 days (10% vs. 7%; *P* = 0.26; adjusted OR: 0.80 [95% CI: 0.42–1.50], *P* = 0.49).

The sensitivity analysis adjusting for procedural complexity (1–2 vs. ≥ 3 passes) confirmed our main results. After this additional adjustment, the odds of achieving an excellent functional outcome did not differ significantly between the mTICI 3 and mTICI 2b groups (adjusted OR: 0.88 [95% CI: 0.59–1.31], *P* = 0.54; Supplementary Table 2).

## Discussion

In this multicenter registry analysis of acute ischemic stroke patients who presented with mild stroke symptoms and anterior circulation large vessel occlusion, we observed that achieving complete reperfusion (mTICI 3) was not associated with improved functional outcomes at 90 days than incomplete reperfusion (mTICI 2b). Functional independence and safety outcomes, including rates of symptomatic intracranial hemorrhage and mortality, were similar between the two reperfusion grades. These findings suggest that, in patients presenting with low NIHSS scores, the added value of pursuing complete reperfusion during endovascular treatment may be limited.

Previous studies in patients with more severe strokes have shown that functional outcomes improve with increasing reperfusion grades [18, 19]. Therefore, the ESO–ESMINT guideline for endovascular therapy endorses mTICI 3 reperfusion, if achievable with reasonable safety [7]. However, our results suggest that this recommendation may not extend to patients with mild stroke symptoms. Several factors may explain the absence of additional benefit from mTICI 3 reperfusion.

First, compared to patients with high baseline NIHSS scores, patients with mild symptoms typically have smaller tissue-at-risk volumes and more robust arterial collaterals. Reperfusion of the proximal major vessels, in combination with robust arterial collaterals, may already be sufficient to reperfuse the smaller penumbra volumes. Therefore, reperfusion of the most distal vessel segments may have limited effect on already well collateralized brain regions [12, 20]. This hypothesis is supported by previous studies, which observed that the clinical benefit of mTICI 3 over mTICI 2b reperfusion progressively decreases with diminishing salvageable penumbra volumes as well as more favorable arterial collaterals [21–23].

On the other hand, in patients with mild stroke, neurological deficits are often determined more by the strategic location of the ischemic lesion, such as involvement of the internal capsule or language-associated cortical areas, than by the infarct volume. Consequently, even complete reperfusion may not restore function when eloquent brain regions have already sustained irreversible injury [24].

Another possible explanation of the observed absent benefit of mTICI 3 reperfusion may lie in the outcome measure of the mRS itself. Although frequently used in stroke research, the mRS may not capture subtle improvements in neurological or cognitive function, that are solely achieved by an mTICI 3 reperfusion compared to mTICI 2b. Thus, even if full reperfusion might lead to small physiological benefits, these may not translate into detectable differences on the mRS score after 90 days [25, 26]. Future studies that include a more granular score to assess neurological deficits in patients with minor stroke, such as the COSMOS scale, are warranted [27].

Furthermore, our data indicate that patients achieving mTICI 2b required a higher number of passes to reach this recanalization grade, which may suggest that these procedures were inherently more challenging. This difficulty might be attributed to underlying anatomical complexities, such as increased arterial tortuosity or elongated vessel segments. Consequently, our findings could imply that for patients with such challenging vascular profiles, it may be prudent to accept an mTICI 2b result as a final outcome, rather than pursuing further maneuvers to achieve mTICI 3.

Although we did not find significant differences in adverse events between patients with mTICI 2b and mTICI 3 reperfusion, additional retrieval attempts to achieve complete reperfusion may increase the risk of endothelial injury or microvascular obstruction, which may not be captured by our dataset. These complications may offset the potential advantage of improved reperfusion status. While our here presented study does not allow recommendations as to whether MT should be continued or stopped as soon as mTICI 2b is achieved, our results suggest that mTICI 2b may represent a clinically acceptable endpoint in selected patients. This is particularly relevant as MT for minor stroke is not yet generally recommended in current American Heart Association (AHA)/American Stroke Association (ASA) [8] or ESO/ESMINT [7] guidelines, where it remains a case-by-case decision due to limited data. In this context, our findings may provide reassurance to clinicians that, in the setting of mild symptoms, the potential procedural risks of pursuing mTICI 3 might outweigh the marginal clinical benefits over a successful mTICI 2b result. Future studies should aim to identify patient subgroups most likely to benefit from achieving mTICI 3 reperfusion, for example by correlating angiographically defined hypoperfused territories with the ischemic core on baseline imaging [22].

The following limitations should be acknowledged: First, it is important to note that the lack of a statistically significant difference in functional outcomes between mTICI 2b and mTICI 3 in our cohort does not establish formal equivalence. Given the observed confidence intervals, we cannot exclude the possibility of a clinical benefit of complete reperfusion that our analysis might have lacked the statistical power to detect. Second, the retrospective design introduces potential selection bias, and unmeasured confounding variables may have influenced clinical outcomes. Perfusion imaging parameters and collateral status were not systematically captured in this registry. While these factors play a role in clinical outcomes, our analysis specifically focused on the impact of the final angiographic reperfusion grade. Future research incorporating detailed imaging data may further refine procedural decision-making during intervention in mild stroke patients. Third, imaging evaluations, including the assessment of mTICI scores, were performed by local investigators at each participating center, which may have introduced interrater variability. Fourth, the final angiographic outcome was evaluated using the mTICI scale, which does not distinguish between TICI 2b and 2c introduced in the expanded TICI (eTICI) score [19, 28]. This categorization may have masked differences in clinical outcomes between incomplete and complete reperfusion. However, prior studies also using the mTICI scale have still shown outcome differences between patients who achieved mTICI 3 and mTICI 2b reperfusion [11, 29].

We focused on the comparison between complete and incomplete reperfusion and excluded patients with failed or minimal reperfusion (mTICI 0–2a). While excluding these cases focuses the analysis on the quality of successful recanalization, it may result in a more favorable outcome profile compared to the total treated mild stroke population.

Lastly, data concerning the presence of a potential intracranial atherosclerotic disease (ICAD), which has been reported to occur more frequently in patients with minor stroke, [30] remain limited. However, an underlying ICAD may also contribute to variations in the final reperfusion status.

## Conclusion

In patients with mild stroke symptoms undergoing MT, no significant differences in functional outcomes were found between patients with complete reperfusion (mTICI 3) and those with incomplete reperfusion (mTICI 2b). These findings suggest that achieving mTICI 2b may represent an adequate final angiographic outcome and highlight the need to carefully reconsider the risk–benefit ratio of additional retrieval attempts aimed at achieving mTICI 3 in this patient population.

## Supplementary Information

ESM1: Supplementary material 1

## Data Availability

Data supporting the findings of this study are available from the corresponding author upon reasonable request after approval of the GSR-ET centers.

## Abbreviations

aOR: adjusted Odds Ratio
ASPECTS: Alberta Stroke Program Early CT Score
CI: Confidence Interval
mTICI: modified Thrombolysis in Cerebral Infarction
ICA: Internal Carotid Artery
IQR: Interquartile Range
mRS: modified Rankin Scale
MT: Mechanical Thrombectomy
NIHSS: National Institutes of Health Stroke Scale
tPA: tissue Plasminogen Activator
