# Supplementary material for: Thrombolysis in Cerebral Infarction (TICI) 2b Versus TICI 3 Reperfusion in Stroke Patients with Mild Symptoms
Source: Clin Neuroradiol. 2026 Mar 13;36(3):879–86. doi: 10.1007/s00062-026-01631-x (PMC13522090; doi:10.1007/s00062-026-01631-x)
Supplement: Supplementary file 1 [file 62_2026_1631_MOESM1_ESM.docx]

Supplementary Material for

**Thrombolysis In Cerebral Infarction (TICI) 2b Versus TICI 3 Reperfusion In Stroke Patients With Mild Symptoms**

Heitkamp et al.

Figure S1. Flow chart of patient inclusion and exclusion criteria.

Table S1. Full model of the multivariable logistic regression analysis of the primary outcome.

Table S2. Multivariable logistic regression analysis including number of passes.

Table S3. German Stroke Registry (GSR) Investigators.

**Figure S1.** Flow chart of patient inclusion and exclusion.

**n = 18069**

included in the GSR-ET (2015 - 2023)

**n = 17322	Excluded**

• other occlusions than ICA, M1 or M2 (n = 5649)

• baseline NIHSS ≥6 (n=11218)

• missing mRS at 90 days (n=208)

• missing prestroke mRS (n=15)

• prestroke mRS ≥2 (n=109)

• mTICI 0-2a (n = 123)

**n = 747**
 patients with an isolated occlusion of the intracranial ICA or of the M1 or M2 segment of the middle cerebral artery and baseline NIHSS <6

**n = 276	mTICI 2b**

**n = 471	mTICI 3**

Included in descriptive statistics

**n = 151	Excluded^*^**

• missing ASPECTS (n =65)

• missing time from symptom onset/last seen well to admission (n = 79)

• missing anesthetic regimen (n = 19)

**n = 596**

Included in the multivariable logistic regression model for the primary outcome

^*^Multiple selection of patients possible.

**Abbreviations:**

GSR-ET: German Stroke Registry-Endovascular Treatment; ICA: Internal Carotid Artery; NIHSS: National Institutes of Health Stroke Scale; mRS: modified Rankin Scale; mTICI: modified Thrombolysis in Cerebral Infarction; CS/LA: Conscious Sedation/Local Anesthesia; GA: General Anesthesia; ASPECTS: Alberta Stroke Program Early CT Score

**Supplementary Table 1.** Full model of the multivariable logistic regression analysis to determine factors independently associated with an excellent functional outcome [90-day mRS score 0-1].

| **Independent variables** | **Adjusted Odds Ratio** | **95% Confidence Interval** | | **p-value** |
| --- | --- | --- | --- | --- |
| mTICI 3 reperfusion [ref. mTICI 2b] | 0.96 | 0.66 | 1.40 | 0.844 |
| Age [per year] | 0.95 | 0.94 | 0.97 | <0.001 |
| Male sex | 1.22 | 0.85 | 1.75 | 0.281 |
| Baseline NIHSS [per point] | 0.86 | 0.77 | 0.97 | 0.012 |
| Time from symptom onset/last seen well to admission [per min.] | 1.00 | 1.00 | 1.00 | 0.095 |
| ASPECTS [per point] | 1.01 | 0.88 | 1.16 | 0.914 |
| ICA/M1 occlusion [ref. M2 occlusion] | 0.93 | 0.65 | 1.34 | 0.711 |
| Administration of tPA | 1.28 | 0.88 | 1.88 | 0.201 |
| General anesthesia [ref. CS/LA] | 0.58 | 0.39 | 0.85 | 0.006 |

596 patients included in multivariable logistic regression model. Statistical significance: *p*<0.05

**Abbreviations:** mRS: modified Rankin Scale; mTICI: modified Thrombolysis in Cerebral Infarction; NIHSS: National Institutes of Health Stroke Scale; ASPECTS: Alberta Stroke Program Early CT Score; ICA: Internal Carotid Artery; tPA: tissue plasminogen activator; CS/LA: Conscious sedation/Local anesthesia

**Supplementary Table 2.** Multivariable logistic regression analysis of factors associated with an excellent functional outcome (mRS 0–1) at 90 days, including number of passes during mechanical thrombectomy.

| **Independent variables** | **Adjusted Odds Ratio** | **95% Confidence Interval** | | **p-value** |
| --- | --- | --- | --- | --- |
| mTICI 3 reperfusion [ref. mTICI 2b] | 0.88 | 0.59 | 1.31 | 0.535 |
| Age [per year] | 0.95 | 0.94 | 0.97 | <0.001 |
| Male sex | 1.16 | 0.80 | 1.70 | 0.426 |
| Baseline NIHSS [per point] | 0.89 | 0.79 | 1.00 | 0.053 |
| Time from symptom onset/last seen well to admission [per min.] | 1.00 | 1.00 | 1.00 | 0.089 |
| ASPECTS [per point] | 0.97 | 0.84 | 1.13 | 0.710 |
| ICA/M1 occlusion [ref. M2 occlusion] | 0.88 | 0.60 | 1.28 | 0.492 |
| Administration of tPA | 1.29 | 0.87 | 1.92 | 0.206 |
| General anesthesia [ref. CS/LA] | 0.59 | 0.40 | 0.89 | 0.011 |
| Number of passes: 1-2 (ref. ≥ 3) | 1.50 | 0.97 | 2.31 | 0.066 |

565 patients included in multivariable logistic regression model. Statistical significance: *p*<0.05

**Abbreviations:** mRS: modified Rankin Scale; mTICI: modified Thrombolysis in Cerebral Infarction; NIHSS: National Institutes of Health Stroke Scale; ASPECTS: Alberta Stroke Program Early CT Score; ICA: Internal Carotid Artery; tPA: tissue plasminogen activator; CS/LA: Conscious sedation/Local anesthesia

**Supplementary Table 3. German Stroke Registry (GSR) Investigators.**

| **Name** | **Location** | **Role** | **Contribution** |
| --- | --- | --- | --- |
| Dr. med. Anna Alegiani | Asklepios Klinik Altona | Site Investigator | Coordination |
| Prof. Dr. med. Jörg Berrouschot | Klinikum Altenburger Land | Site Investigator | Coordination |
| PD Dr. med. Tobias Boeckh-Behrens | Klinikum rechts der Isar der Technischen Universität München | Site Investigator | Coordination |
| Dr.med. Georg Bohner | Charité Berlin | Site Investigator | Coordination |
| Prof. Dr. med. Jan Borggrefe | Johannes Wesling Klinikum Minden | Site Investigator | Coordination |
| Dr. med. Albrecht Bormann | Klinikum Altenburger Land | Site Investigator | Coordination |
| Dr. med. Michael Braun | Bezirkskrankenhaus Günzburg | Site Investigator | Coordination |
| Prof. Dr. med. Franziska Dorn | Universitätsklinikum Bonn | Site Investigator | Coordination |
| Prof. Dr. med. Bernd Eckert | Asklepios Hamburg Altona | Site Investigator | Coordination |
| Prof. Dr. med. Ulrike Ernemann | Universitätsklinik Tübingen | Site Investigator | Coordination |
| PD Dr. med. Marielle Ernst | Georg-August-Universität Göttingen | Site Investigator | Coordination |
| Prof. Dr. med. Jens Fiehler | UKE Hamburg-Eppendorf | Site Investigator | Coordination |
| Prof. Dr. med. Christian Gerloff | UKE Hamburg-Eppendorf | Site Investigator | Coordination |
| Prof. Dr. med. Klaus Gröschel | Uniklinik Mainz | Site Investigator | Coordination |
| Prof. Dr. med. Gerhard F. Hamann | Bezirkskrankenhaus Günzburg | Site Investigator | Coordination |
| Dr. med. Jörg Hattingen | Klinikum Nordstadt | Site Investigator | Coordination |
| Dr. med. Karl-Heinz Henn | Klinikum Offenbach | Site Investigator | Coordination |
| Dr. med. Fee Keil | Uniklinik Frankfurt/Main | Site Investigator | Coordination |
| Prof. Dr. med. Lars Kellert | LMU Klinikum | Site Investigator | Coordination |
| Dr. med. Christoffer Kraemer | Klinikum Lüneburg | Site Investigator | Coordination |
| PD Dr. med. Ruben Mühl-Benninghaus | Klinikum Lüneburg | Site Investigator | Coordination |
| Prof. Dr. med. Jan Liman | Georg-August-Universität Göttingen | Site Investigator | Coordination |
| Dr. med. Alexander Ludolph | Klinikum Offenbach | Site Investigator | Coordination |
| Prof. Dr. med. Christian Nolte | Charité Berlin | Site Investigator | Coordination |
| Prof. Dr. med. Omid Nikoubashman | Uniklinik RWTH Aachen | Site Investigator | Coordination |
| Dr. med. Martina Petersen | Klinikum Osnabrück | Site Investigator | Coordination |
| Prof. Dr. med. Gabor Petzold | Universitätsklinikum Bonn | Site Investigator | Coordination |
| PD Dr. med. Sven Poli | Universitätsklinik Tübingen | Site Investigator | Coordination |
| PD Dr. med. Arno Reich | Uniklinik RWTH Aachen | Site Investigator | Coordination |
| Prof. Dr. med. Joachim Röther | Asklepios Hamburg Altona | Site Investigator | Coordination |
| Dr. med. Jan Hendrik Schäfer | Uniklinik Frankfurt/Main | Site Investigator | Coordination |
| Maximilian Schell | UKE Hamburg-Eppendorf | Site Investigator | Coordination |
| Prof. Dr. med. Peter Schellinger | Johannes Wesling Klinikum Minden | Site Investigator | Coordination |
| PD Dr. med Eberhard Siebert | Charité Berlin | Site Investigator | Coordination |
| Prof. Dr. med. Florian Stögbauer | Klinikum Osnabrück | Site Investigator | Coordination |
| Prof. Dr. med. Götz Thomalla | UKE Hamburg-Eppendorf | Site Investigator | Coordination |
| Dr. med. Steffen Tiedt | LMU Klinikum | Site Investigator | Coordination |
| Prof. Dr. med. Christoph Trumm | LMU Klinikum | Site Investigator | Coordination |
| Dr. med. Timo Uphaus | Uniklinik Mainz | Site Investigator | Coordination |
| Dr. med. Silke Wunderlich | Klinikum rechts der Isar der Technischen Universität München | Site Investigator | Coordination |
| Dr. med. Sarah Zweynert | Charité Berlin | Site Investigator | Coordination |
